# Potassium binders and continuation of renin–angiotensin system inhibitors/mineralocorticoid receptor antagonist in chronic kidney disease and heart failure (the DEMONSTRATE database)

**DOI:** 10.1111/joim.70087

**Published:** 2026-03-26

**Authors:** Hans Furuland, Anders Olof Larsson, Milica Uhde, Kristel Parv, Matilda Almstedt, Thomas Cars, Maria K. Svensson

**Affiliations:** ^1^ Department of Medical Sciences, Renal Medicine Uppsala University Hospital Uppsala Sweden; ^2^ Department of Medical Sciences, Clinical Chemistry Uppsala University Hospital Uppsala Sweden; ^3^ Vifor Pharma Nordiska AB Solna Sweden; ^4^ Sence Research AB Uppsala Sweden; ^5^ Uppsala Clinical Research Center Uppsala Sweden

**Keywords:** hyperkalemia, mineralocorticoid receptor antagonists, potassium binders, renin–angiotensin system inhibitors, treatment persistence

## Abstract

**Introduction:**

Hyperkalaemia is a serious complication in patients with chronic kidney disease (CKD) and heart failure (HF), often leading to the discontinuation of renin–angiotensin–aldosterone system inhibitors (RAASi; renin‐angiotensin system inhibitors [RASi] and mineralocorticoid receptor antagonists [MRAs]) despite their cardiorenal benefits. Although potassium binders, especially second‐generation potassium binders, reduce the risk of hyperkalaemia in clinical trials, real‐world evidence on whether they enable RAASi continuation and influence clinical outcomes remains scarce.

**Methods:**

This observational, population‐based cohort study used Swedish healthcare data from the DEMONSTRATE database to identify adults with non‐dialysis (ND) CKD and/or HF who initiated potassium binders (2018–2022). Patients were stratified by potassium binder generation. RASi and MRA treatment changes were assessed at 6 months, and clinical outcomes (all‐cause mortality, hospitalization and three‐point major adverse cardiovascular events [3P‐MACE]) were evaluated over a 3‐year follow‐up using propensity‐score‐weighted Kaplan–Meier analyses.

**Results:**

Of 7913 potassium binder episodes (6232 patients), 7.1% used second‐generation binders. These episodes were associated with increased RAASi persistence at 6 months: 76.9% and 57.5% of second‐generation users maintained RASi and MRA therapy, respectively, compared with 66.4% and 47.8% in the first‐generation group. Patients who maintained RASi had lower observed all‐cause mortality and hospitalization rates, but no difference in incidence of 3P‐MACE was found.

**Conclusion:**

Second‐generation potassium binders were associated with higher RAASi persistence than first‐generation binders. RASi persistence was associated with lower observed rates of all‐cause mortality and hospitalization, with no clear differences in 3P‐MACE. These findings suggest that potassium binders may enable sustained RAASi use in patients with ND‐CKD and/or HF.

## Graphical abstract

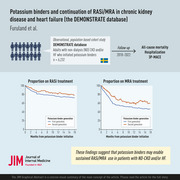

**Abbreviations**
3P‐MACE: three‐point major adverse cardiovascular events
ACEi: angiotensin‐converting enzyme inhibitor
ARB: angiotensin receptor blocker
ARNi: angiotensin receptor–neprilysin inhibitors
ATC: Anatomical Therapeutic Chemical
CKD: chronic kidney disease
COVID‐19: coronavirus disease 2019
eGFR: estimated glomerular filtration rate
HF: heart failure
ICD‐10: International Classification of Diseases, 10th Revision
IHD: ischaemic heart disease
IQR: interquartile range
MACE: major adverse cardiovascular events
MRA: mineralocorticoid receptor antagonists
ND‐CKD: non‐dialysis chronic kidney disease
RAASi: renin–angiotensin–aldosterone system inhibitors
RASi: renin–angiotensin system inhibitors
SGLT2i: sodium–glucose cotransporter 2 inhibitors
SMD: standardized mean difference
SPS: sodium polystyrene sulphonate

## Introduction

Renin–angiotensin–aldosterone system inhibitors (RAASi) are essential therapies for cardiorenal protection in patients with chronic kidney disease (CKD), diabetes mellitus and cardiovascular disease, particularly heart failure (HF). Despite their well‐established benefits in improving long‐term cardiorenal outcomes, RAASi use further increases the elevated risk of hyperkalaemia in these patient populations [1, 2, 3, 4, 5, 6]. Hyperkalaemia, defined as serum potassium levels exceeding 5.0 mmol/L, may lead to life‐threatening cardiac arrhythmias and increased risk of mortality [4]. Although temporary discontinuation or down‐titration of RAASi treatment is recommended in the event of a hyperkalaemia episode, permanent discontinuation of treatment is common [1, 7, 8, 9, 10, 11]. Managing hyperkalaemia is essential to enable treatment with maximum tolerated guideline‐directed dosage of RAASi and reduces long‐term morbidity and mortality in these patients [5, 6].

Second‐generation potassium binders, patiromer and sodium zirconium cyclosilicate, have recently received regulatory approval for the management of hyperkalaemia, mainly due to their improved safety profiles compared with the first‐generation binder sodium polystyrene sulphonate (SPS) [7, 12]. Clinical trials have shown that second‐generation potassium binders reduce the risk of hyperkalaemia and support continued use and optimization of RAASi therapy [9, 13, 14, 15]. However, real‐world evidence is limited on how potassium binders, especially second‐generation agents, influence clinical decision‐making on RAASi continuation and affect long‐term outcomes in high‐risk patients treated with potassium binders.

This nationwide observational study in Sweden was conducted within the DEMONSTRATE database utilizing comprehensive regional and national data [16]. The study aimed at examining whether the use of second‐generation potassium binders was associated with continued RAASi therapy in patients with non‐dialysis CKD (ND‐CKD), HF or both. Additionally, we explored associations between maintained RAASi use and clinical outcomes, including major adverse cardiovascular events (MACE), all‐cause hospitalization and mortality.

## Methods

### Data source

This observational, population‐based cohort study was conducted in Sweden, where all residents have access to a comprehensive nationwide public healthcare system characterized by minimal co‐payments for healthcare visits, hospitalizations and medications [17]. Each resident's interactions with the healthcare system, including the filling of prescriptions, are recorded using a unique personal identification number, providing nearly complete population‐wide medical history.

The analysis was based on data from the DEMONSTRATE research project, which integrates information from multiple national and regional sources, including the National Patient Register, the Swedish Prescribed Drug Register [18], the Cause of Death Register [19], regional electronic medical records and clinical chemistry databases. Individual‐level data from these sources were linked through the unique personal identification number, pseudonymized for privacy and consolidated into the DEMONSTRATE database [16].

The study was conducted in accordance with the Declaration of Helsinki and was approved by the Swedish Ethical Review Authority (approval numbers 2022‐01051‐01 and 2022‐06306‐02). Given the nature of the study, informed consent was not required.

### Patients

The study sample comprised all individuals aged 18 years or older in Sweden with a diagnosis of CKD and/or HF who initiated treatment with a potassium binder between 1 January 2018 and 31 December 2022. The study start date was chosen when the second‐generation potassium binders became available for prescription in Sweden. The study included both first‐generation potassium binders, that is, SPS, and second‐generation agents, that is, patiromer and sodium zirconium cyclosilicate. The index date was defined as the start of a potassium binder treatment episode. Patients were excluded if they had undergone dialysis on or before the index date, or if they had less than 6 months of follow‐up data. Minimum of 6‐month follow‐up was required, as this time point was used to classify changes in renin‐angiotensin system inhibitor (RASi) and mineralocorticoid receptor antagonist (MRA) treatment. All variable definitions and algorithms for treatment episode construction are provided in detail in the Supporting Information section.

### Patient characteristics

Diagnoses and clinical procedure codes from both inpatient and outpatient care were used to identify prevalent comorbidities other than CKD and/or HF, based on all available data up to and including the index date. Diagnoses were classified using the International Classification of Diseases, 10th Revision (ICD‐10) [20]. Medication use was defined as at least one dispensed prescription within the 6‐month preceding and including the index date, based on Anatomical Therapeutic Chemical (ATC) codes [21]. All code lists and definitions are provided in the Supporting Information section.

### Outcomes

The study outcomes included the continuation of RASi and/or MRA therapy, following the initiation of potassium binder treatment, as well as clinical outcomes: all‐cause mortality, all‐cause hospitalization and three‐point major adverse cardiovascular events (3P‐MACE; cardiovascular death, myocardial infarction or stroke).

### Definitions

#### Changes in renin–angiotensin–aldosterone system inhibitor use

To assess changes in the use of RASi and MRA therapy following the initiation of potassium binder treatment, we included patients who had filled at least one prescription for a RASi, that is, angiotensin‐converting enzyme inhibitor or angiotensin receptor blocker or an MRA within the 6 months prior to or on the index date. Treatment changes were evaluated over a 12‐month period spanning 6 months before to 6 months after the index date and were categorized as maintenance, down‐titration or discontinuation. The most recent dispensation prior to the index date was used to define the reference dose for each drug class (baseline). The dispensation closest to the 6 months after index was used to define the treatment change. Down‐titration was defined as a reduction in the prescribed daily dose at 6 months post‐index compared with baseline. Discontinuation was defined as the absence of medical supply at 6 months after the index date, allowing for a 25% grace period on previous dispensations to account for non‐adherence. Maintenance was defined as the absence of both dose reduction and discontinuation. RASi and MRA were analysed separately, and non‐steroidal MRA was not available during the study period (further details are provided in the Supporting Information section). As the choice of a 6‐month classification window involves a trade‐off between allowing sufficient time for stable prescribing patterns and the risk of misclassifying patients who change treatment early, a sensitivity analysis using a 4‐month window was performed for the outcome of all‐cause mortality.

### Statistical analysis

Descriptive statistics were used to summarize baseline patient characteristics. Continuous variables were reported as medians with interquartile ranges (IQRs), as appropriate. Categorical variables were presented as frequencies and percentages. Classification into first‐ or second‐generation potassium binder episodes was based on the generation of binder used at treatment initiation.

#### Temporal dynamics of RASi and MRA treatment in relation to potassium binder initiation

To describe the proportion of patients receiving RASi and/or MRA therapy following the initiation of potassium binder treatment, we assessed an 18‐month follow‐up period starting from the index date. For each day during this period, we calculated the proportion of patients with an active supply of RASi and/or MRA (numerator) relative to the number of patients remaining under follow‐up on that day (denominator). The analysis was stratified by the generation of potassium binder. In a supplementary analysis, we also evaluated the proportion of patients receiving RASi and/or MRA during the 18 months preceding potassium binder initiation. To be included in either analysis, treatment episodes were required to have an active supply of RASi or MRA at the index date. These figures present unadjusted data to illustrate treatment patterns as observed in clinical practice; adjusted comparisons are provided in the outcome analyses.

#### Clinical outcomes

For the analysis of all‐cause mortality, all‐cause hospitalization and 3P‐MACE, that is, cardiovascular death, myocardial infarction or stroke, the study sample was limited to patients with documented use of RASi and/or MRA at baseline. Follow‐up began 6 months after the index date, the time point used to classify changes in RASi and MRA treatment. Patients were followed until the first occurrence of the outcome of interest, death, start of a subsequent potassium binder episode or the end of follow‐up (31 December 2022), with a maximum duration of 3 years. Analyses were conducted stratified by (a) RASi/MRA treatment change, (b) potassium binder generation and (c) combined categories of RASi/MRA treatment change and potassium binder generation. For the potassium binder generation comparisons, the analysis was restricted to episodes with index date from 2019 onwards to ensure sufficient overlap between treatment groups, as second‐generation binders were rarely prescribed before 2019.

For each outcome, Kaplan–Meier curves were generated both unweighted and weighted using propensity score weights, with 95% confidence interval bands. Hazard ratios with 95% confidence intervals were estimated using Cox proportional hazards regression.

The proportional hazards assumption was assessed by a visual inspection of Schoenfeld residuals for the three main RASi‐based analyses. Visual inspection suggested minor departures from proportionality; we determined that the reported hazard ratios represent valid summaries of the average effects across the study period.

The weighted analyses were performed to reduce confounding by balancing observed covariates between exposure groups. Separate propensity score models were estimated for each comparison (six primary models: three for RASi and three for MRA), with two additional models examining RASi/MRA changes stratified by potassium binder generation included as sensitivity analyses. Propensity scores were estimated using logistic regression, with covariates selected based on clinical relevance and expert input from specialists in cardiovascular and renal medicine. The following baseline covariates were included in all models: age, sex, presence of CKD and/or HF (including time since diagnosis), hypertension, Type I and II diabetes mellitus, ischaemic heart disease (IHD), prior inpatient healthcare utilization, concomitant cardiovascular medications (beta‐blockers, diuretics, calcium channel blockers, sodium–glucose cotransporter 2 inhibitors [SGLT2i] and angiotensin receptor–neprilysin inhibitors [ARNi]) and calendar year. Prior to weighting, extreme weights were truncated at the 99th percentile [22]. Covariate balance between groups was evaluated using standardized mean differences (SMD) [23], with an SMD < 0.1 considered indicative of adequate balance. Propensity score weights reflecting the average treatment effect were then derived from the predicted probabilities. Robust (sandwich) standard errors were used to account for clustering of multiple episodes within patients and to provide valid inference when using estimated propensity score weights. In a sensitivity analysis, baseline estimated glomerular filtration rate (eGFR) and potassium were added to the propensity score model; for patients with missing laboratory values, multiple imputations were used (10 imputed datasets, pooled according to Rubin's rules). For the outcome of all‐cause mortality, we also reported the most common underlying causes of death. For hospitalization, the most frequently recorded primary diagnoses (ICD‐code) were described to provide clinical context for the observed events.

All analyses were conducted using R version 4.3.3.

## Results

The study sample comprised 7913 potassium binder treatment episodes among 6232 patients with CKD and/or HF, no prior history of dialysis and a minimum follow‐up of 6 months (mean follow‐up time 1.59 [SD: 1.07 years]; Fig. S1). The median patient age was 74.0 years (IQR: 64.0–81.0), and 68.5% were male. The majority of episodes (*n* = 7350; 92.9%) involved first‐generation potassium binders, whereas second‐generation agents were used in 563 episodes (7.1%; Table 1). At the time of potassium binder initiation, 77.2% of episodes (*n* = 6108) were associated with ongoing treatment with RASi and 18.9% (*n* = 1496) with MRA.

**Table 1:** Baseline characteristics of potassium binder treatment episodes, stratified by potassium binder generation.

Variable	Total (all potassium binder episodes)	First‐generation potassium binders	Second‐generation potassium binders
Number of potassium binder episodes, *N* (%)	7913 (100%)	7350 (92.9%)	563 (7.1%)
Number of patients	6232	5830	505
Demographics			
Age at index, median (IQR)	74.0 (64.0–81.0)	74.0 (64.0–82.0)	72.0 (59.0–79.0)
Male, *n* (%)	5417 (68.5%)	5040 (68.6%)	377 (67.0%)
Female, *n* (%)	2496 (31.5%)	2310 (31.4%)	186 (33.0%)
HF, *n* (%)	3202 (40.5%)	2934 (39.9%)	268 (47.6%)
CKD, *n* (%)	6717 (84.9%)	6268 (85.3%)	449 (79.8%)
– HF only	1196 (15.1%)	1082 (14.7%)	114 (20.2%)
– CKD only	4711 (59.5%)	4416 (60.1%)	295 (52.4%)
– HF and CKD	2006 (25.4%)	1852 (25.2%)	154 (27.4%)
Comorbid conditions, *n* (%)			
Hypertension	7004 (88.5%)	6518 (88.7%)	486 (86.3%)
Ischaemic heart disease	2597 (32.8%)	2407 (32.7%)	190 (33.7%)
Diabetes mellitus (any type)	3707 (46.8%)	3443 (46.8%)	264 (46.9%)
Diabetes mellitus Type I	555 (7.0%)	494 (6.7%)	61 (10.8%)
Diabetes mellitus Type II	3126 (39.5%)	2924 (39.8%)	202 (35.9%)
Type of potassium binder, *n* (%)			
1st generation	7350 (92.9%)	7350 (100.0%)	N/A
2nd generation	563 (7.1%)	N/A	563 (100.0%)
Potassium binder at 6 months post index	2755 (34.8%)	2502 (34.0%)	253 (44.9%)
Medications, *n* (%)			
Ongoing RASi treatment	6108 (77.2%)	5623 (76.5%)	485 (86.1%)
Ongoing MRA treatment	1496 (18.9%)	1315 (17.9%)	181 (32.1%)
Ongoing RASi and MRA treatment	1354 (17.1%)	1181 (16.1%)	173 (30.7%)
Diuretics^a^	4590 (58.0%)	4248 (57.8%)	342 (60.7%)
Beta blockers^a^	5546 (70.1%)	5123 (69.7%)	423 (75.1%)
Calcium channel blockers^a^	4351 (55.0%)	4089 (55.6%)	262 (46.5%)
ARNi^a^	282 (3.6%)	200 (2.7%)	82 (14.6%)
SGLT‐2i^a^	280 (3.5%)	216 (2.9%)	64 (11.4%)

Abbreviations: ACEi, angiotensin‐converting enzyme inhibitors; ARBs, angiotensin receptor blockers; ARNi, angiotensin receptor–neprilysin inhibitors; CKD, chronic kidney disease; HF, heart failure; IQR, interquartile range; MRA, mineralocorticoid receptor antagonists; RASi, renin–angiotensin system inhibitors; SGLT2i, sodium–glucose cotransporter 2 inhibitors.

^a^Within180 days prior to index (including the index date).

Compared with episodes with first‐generation potassium binders, those treated with second‐generation agents were associated with younger age (median 72.0 years [IQR: 59.0–79.0] vs. 74.0 years [IQR: 64.0–82.0]) (Table 1). Additionally, there was a higher prevalence of HF (47.6% vs. 39.9%) and Type I diabetes (10.8% vs. 6.7%) in the second‐generation group. The proportion of patients with both ND‐CKD and HF was also higher in the second‐generation group (27.4% vs. 25.2%), whereas CKD only was less frequent (52.4% vs. 60.1%).

Second‐generation potassium binder users were more likely to have treatment with RASi (86.1% vs. 76.5%) and MRA (32.1% vs. 17.9%) at index. Use of contemporary therapies for cardiorenal disease, including ARNi (14.6% vs. 2.7%) and SGLT2i (11.4% vs. 2.9%), was also more common in the second‐generation group. Furthermore, a higher proportion of patients treated with second‐generation potassium binders remained on potassium binder therapy at 6 months post‐initiation compared with those receiving first‐generation agents (44.9% vs. 34.0%).

**Fig. 1:**
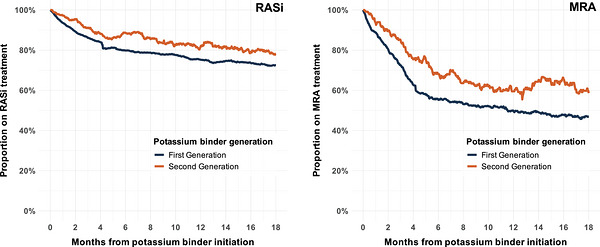
Proportion of patients remaining on RASi and MRA therapy following initiation of potassium binder treatment. MRA, mineralocorticoid receptor antagonists; RASi, renin‐angiotensin system inhibitors.

Fig. 1 shows the proportion of patients receiving RASi or MRA therapy over time following potassium binder initiation, stratified by potassium binder generation, that is, first‐ or second‐generation. This analysis focuses on treatment status over time rather than dose adjustments or discontinuations. Throughout the 18‐month follow‐up, a more sustained use of both RASi and MRA was observed among patients on second‐generation binders. Although both groups showed an initial decline in use after initiation, treatment proportions stabilized at higher levels among second‐generation users, with a persistent separation from first‐generation episodes over time. In a supplementary analysis, we also examined RASi and MRA use during the 18 months preceding potassium binder initiation. A slightly higher proportion of patients were on both RASi and MRA prior to the index date in the first‐generation group compared with the second‐generation group (Fig. S2).

In the analysis of subsequent changes in RASi treatment status, a higher proportion of patients in the second‐generation potassium binder group maintained RASi therapy during the 6 months following initiation compared with the first‐generation group (76.3% vs. 66.4%) (Table 2a). Treatment discontinuation was observed less frequently among second‐generation users (13.8% vs. 22.2%), whereas the proportion of patients with dose down‐titration was similar between groups (9.9% vs. 11.5%). A similar, though less pronounced, difference was observed for MRA treatment, with 57.5% of second‐generation binder users maintaining therapy compared with 48.1% in the first‐generation group (Table 2b).

**Table 2a:** Baseline characteristics of potassium binder episodes with RASi use at baseline, stratified by subsequent change in RASi treatment.

		First‐generation potassium binder				Second‐generation potassium binder			
	All potassium binder episodes with baseline RASi use	All first‐generation episodes with baseline RASi use	Maintained RASi	Down‐titrated RASi	Discontinued RASi	All second‐generation episodes with baseline RASi use	Maintained RASi	Down‐titrated RASi	Discontinued RASi
Number of episodes, *N* (%)	6108	5623 (100)	3732 (66.4)	645 (11.5)	1246 (22.2)	485 (100)	370 (76.3)	48 (9.9)	67 (13.8)
Number of patients	4920	4556	3033	631	1211	438	334	48	66
*Demographics*									
Age at index, median (IQR)	73.0 (63.0–81.0)	73.0 (63.0–81.0)	73.0 (62.0–80.0)	74.0 (64.0–81.0)	76.0 (66.0–83.0)	72.0 (59.0–79.0)	72.0 (59.0–79.0)	74.0 (62.0–78.0)	71.0 (60.0–79.0)
Male, *n* (%)	4236 (69.4)	3911 (69.6)	2609 (69.9)	447 (69.3)	855 (68.6)	325 (67.0)	252 (68.1)	29 (60.4)	44 (65.7)
Female, *n* (%)	1872 (30.6)	1712 (30.4)	1123 (30.1)	198 (30.7)	391 (31.4)	160 (33.0)	118 (31.9)	19 (39.6)	23 (34.3)
*Comorbid conditions, n (%)*									
HF	2653 (43.4)	2410 (42.9)	1505 (40.3)	339 (52.6)	566 (45.4)	243 (50.1)	181 (48.9)	28 (58.3)	34 (50.7)
CKD	5028 (82.3)	4652 (82.7)	3135 (84.0)	485 (75.2)	1032 (82.8)	376 (77.5)	289 (78.1)	36 (75.0)	51 (76.1)
‐ HF only	1080 (17.7)	971 (17.3)	597 (16.0)	160 (24.8)	214 (17.2)	109 (22.5)	81 (21.9)	12 (25.0)	16 (23.9)
‐ CKD only	3455 (56.6)	3213 (57.1)	2227 (59.7)	306 (47.4)	680 (54.6)	242 (49.9)	189 (51.1)	20 (41.7)	33 (49.3)
‐HF and CKD	1573 (25.8)	1439 (25.6)	908 (24.3)	179 (27.8)	352 (28.3)	134 (27.6)	100 (27.0)	16 (33.3)	18 (26.9)
Duration of HF (years), median (IQR)	3.8 (1.2–8.2)	3.8 (1.2–8.1)	3.8 (1.2–8.2)	4.2 (1.3–8.4)	3.6 (1.1–7.5)	3.4 (1.0–9.8)	3.4 (1.1–9.4)	4.9 (1.5–10.5)	2.3 (0.6–7.5)
Duration of CKD (years), median (IQR)	4.5 (1.7–8.9)	4.5 (1.7–8.8)	4.9 (2.0–9.2)	4.2 (1.2–8.5)	3.8 (1.1–7.9)	4.1 (1.3–9.3)	4.1 (1.4–9.3)	3.5 (0.8–7.7)	4.6 (2.2–11.5)
Hypertension	5507 (90.2)	5088 (90.5)	3383 (90.6)	582 (90.2)	1123 (90.1)	419 (86.4)	321 (86.8)	43 (89.6)	55 (82.1)
Ischaemic heart disease	2108 (34.5)	1936 (34.4)	1234 (33.1)	244 (37.8)	458 (36.8)	172 (35.5)	126 (34.1)	21 (43.8)	25 (37.3)
Diabetes Mellitus (any)	3014 (49.3)	2782 (49.5)	1864 (49.9)	313 (48.5)	605 (48.6)	232 (47.8)	174 (47.0)	23 (47.9)	35 (52.2)
Diabetes Mellitus Type I	479 (7.8)	425 (7.6)	307 (8.2)	46 (7.1)	72 (5.8)	54 (11.1)	39 (10.5)	7 (14.6)	8 (11.9)
Diabetes Mellitus Type II	2513 (41.1)	2336 (41.5)	1543 (41.3)	264 (40.9)	529 (42.5)	177 (36.5)	134 (36.2)	16 (33.3)	27 (40.3)
*Potassium binder, n (%)*									
Potassium binder at 6 months post index	2149 (35.2)	1922 (34.2)	1423 (38.1)	196 (30.4)	303 (24.3)	227 (46.8)	183 (49.5)	23 (47.9)	21 (31.3)
*Medications, n (%)*									
Diuretics^a^	3724 (61.0)	3422 (60.9)	2200 (58.9)	427 (66.2)	795 (63.8)	302 (62.3)	221 (59.7)	36 (75.0)	45 (67.2)
Beta blockers^a^	4374 (71.6)	4008 (71.3)	2607 (69.9)	489 (75.8)	912 (73.2)	366 (75.5)	286 (77.3)	34 (70.8)	46 (68.7)
Calcium channel blockers^a^	3393 (55.6)	3171 (56.4)	2104 (56.4)	353 (54.7)	714 (57.3)	222 (45.8)	170 (45.9)	20 (41.7)	32 (47.8)
ARNi^a^	282 (4.6)	200 (3.6)	144 (3.9)	36 (5.6)	20 (1.6)	82 (16.9)	70 (18.9)	8 (16.7)	<=5
SGLT‐2i^a^	261 (4.3)	200 (3.6)	145 (3.9)	26 (4.0)	29 (2.3)	61 (12.6)	53 (14.3)	6 (12.5)	<=5

Abbreviations: ACEi, angiotensin‐converting enzyme inhibitors; ARBs, angiotensin receptor blockers; ARNi, angiotensin receptor–neprilysin inhibitors; CKD, chronic kidney disease; HF, heart failure; IQR, interquartile range; MRA, mineralocorticoid receptor antagonists; RASi, renin–angiotensin system inhibitors; SGLT2i, sodium–glucose cotransporter 2 inhibitors.

^a^ Within180 days prior to index (including the index date).

**Table 2b:** Baseline characteristics of potassium binder episodes with MRA use at baseline, stratified by subsequent change in MRA treatment.

		First‐generation potassium binder				Second‐generation potassium binder			
	All potassium binder episodes with baseline MRA use	All first generation episodes with baseline MRA use	Maintained MRA	Down‐titrated MRA	Discontinued MRA	All second generation episodes with baseline MRA use	Maintained MRA	Down‐titrated MRA	Discontinued MRA
Number of episodes, *N* (%)	1496	1315 (100)	633 (48.1)	71 (5.4)	611 (46.5)	181 (100)	104 (57.5)	16 (8.8)	61 (33.7)
Number of patients	1349	1189	564	71	601	167	97	16	60
*Demographics*									
Age at index. median (IQR)	76.0 (70.0–83.0)	76.0 (70.0–83.0)	76.0 (70.0–82.0)	75.0 (71.0–82.0)	77.0 (71.0–84.0)	75.0 (68.0–80.0)	75.0 (67.0–81.0)	76.5 (72.0–83.0)	74.0 (67.0–78.0)
Male, *n* (%)	929 (62.1)	814 (61.9)	398 (62.9)	42 (59.2)	374 (61.2)	115 (63.5)	61 (58.7)	10 (62.5)	44 (72.1)
Female, *n* (%)	567 (37.9)	501 (38.1)	235 (37.1)	29 (40.8)	237 (38.8)	66 (36.5)	43 (41.3)	6 (37.5)	17 (27.9)
*Comorbid conditions, n (%)*									
HF	1226 (82.0)	1079 (82.1)	511 (80.7)	56 (78.9)	512 (83.8)	147 (81.2)	83 (79.8)	14 (87.5)	50 (82.0)
CKD	826 (55.2)	725 (55.1)	372 (58.8)	34 (47.9)	319 (52.2)	101 (55.8)	64 (61.5)	7 (43.8)	30 (49.2)
‐ HF only	670 (44.8)	590 (44.9)	261 (41.2)	37 (52.1)	292 (47.8)	80 (44.2)	40 (38.5)	9 (56.2)	31 (50.8)
‐ CKD only	270 (18.0)	236 (17.9)	122 (19.3)	15 (21.1)	99 (16.2)	34 (18.8)	21 (20.2)	2 (12.5)	11 (18.0)
‐ HF and CKD	556 (37.2)	489 (37.2)	250 (39.5)	19 (26.8)	220 (36.0)	67 (37.0)	43 (41.3)	5 (31.2)	19 (31.1)
Duration of HF (years)	3.7 (1.1–7.9)	3.8 (1.2–7.9)	3.6 (1.1–7.2)	4.4 (1.6–8.9)	4.0 (1.2–8.0)	3.0 (0.7–9.6)	3.2 (1.0–9.1)	4.1 (1.5–12.2)	1.8 (0.5–9.3)
Duration of CKD (years)	2.8 (0.9–6.1)	2.8 (0.9–6.0)	2.9 (1.1–6.0)	2.7 (1.1–6.5)	2.8 (0.6–5.8)	2.5 (0.6–7.9)	2.5 (0.6–7.5)	1.7 (0.7–5.7)	2.7 (0.6–8.4)
Hypertension	1310 (87.6)	1154 (87.8)	557 (88.0)	63 (88.7)	534 (87.4)	156 (86.2)	93 (89.4)	14 (87.5)	49 (80.3)
Ischaemic heart disease	751 (50.2%)	658 (50.0%)	320 (50.6%)	32 (45.1%)	306 (50.1%)	93 (51.4%)	52 (50.0%)	7 (43.8%)	34 (55.7%)
Diabetes mellitus (any)	757 (50.6)	661 (50.3)	315 (49.8)	39 (54.9)	307 (50.2)	96 (53.0)	53 (51.0)	6 (37.5)	37 (60.7)
Diabetes mellitus Type I	74 (4.9)	59 (4.5)	30 (4.7)	<=5	27 (4.4)	15 (8.3)	9 (8.7)	<=5	<=5
Diabetes mellitus Type II	678 (45.3)	597 (45.4)	283 (44.7)	37 (52.1)	277 (45.3)	81 (44.8)	44 (42.3)	<=5	32 (52.5)
*Potassium binder, n (%)*									
Potassium binder at 6 months post index	403 (26.9)	324 (24.6)	208 (32.9)	18 (25.4)	98 (16.0)	79 (43.6)	51 (49.0)	8 (50.0)	20 (32.8)
*Medications, n (%)*									
Diuretics^a^	1116 (74.6)	989 (75.2)	479 (75.7)	51 (71.8)	459 (75.1)	127 (70.2)	67 (64.4)	12 (75.0)	48 (78.7)
Beta blockers^a^	1343 (89.8)	1178 (89.6)	570 (90.0)	63 (88.7)	545 (89.2)	165 (91.2)	96 (92.3)	16 (100.0)	53 (86.9)
Calcium channel blockers^a^	522 (34.9)	474 (36.0)	224 (35.4)	26 (36.6)	224 (36.7)	48 (26.5)	28 (26.9)	<=5	18 (29.5)
ARNi^a^	194 (13.0)	134 (10.2)	69 (10.9)	9 (12.7)	56 (9.2)	60 (33.1)	31 (29.8)	8 (50.0)	21 (34.4)
SGLT‐2i^a^	136 (9.1)	98 (7.5)	49 (7.7)	9 (12.7)	40 (6.5)	38 (21.0)	22 (21.2)	<=5	15 (24.6)

Abbreviations: ACEi, angiotensin converting enzyme inhibitors; ARBs, Angiotensin Receptor Blockers; ARNi, angiotensin receptor‐neprilysin inhibitors; CKD, chronic kidney disease; HF, heart failure; IQR, interquartile range; MRA, mineralocorticoid receptor antagonists; RASi renin‐angiotensin system inhibitors; SGLT2i, sodium‐glucose cotransporter 2 inhibitors.

^a^ Within180 days prior to index (including the index date).

**Fig. 2:**
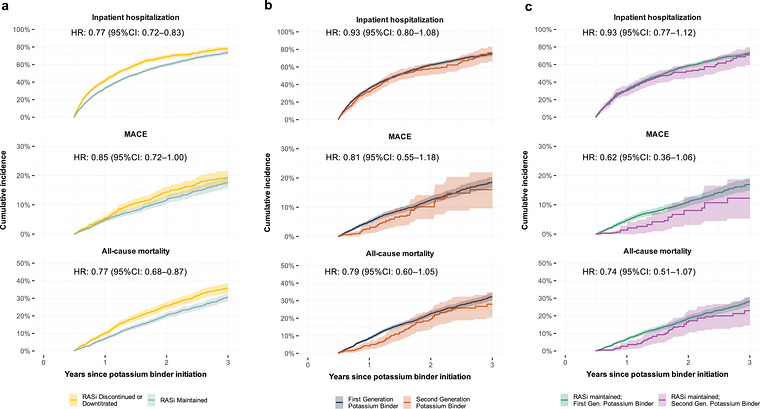
Weighted cumulative incidence of clinical outcomes stratified by (a) change in RASi treatment, (b) potassium binder generation, and (c) combined categories of RASi treatment change and potassium binder generation. Weighted Kaplan–Meier estimates for clinical outcomes among episodes with baseline RASi use. Corresponding unweighted estimates, as well as estimates for MRA treatment change, are shown in Supplementary Figs. Estimates for RASi and MRA discontinuation stratified by potassium binder generation are presented in the Supplementary. MACE, major adverse cardiovascular event; RASi, renin‐angiotensin system inhibitors.

During follow‐up, a total of 3111 inpatient episodes (70.9/1000 patient‐years), 1195 deaths (16.1/1000 patient‐years) and 623 MACE events (8.6/1000 patient‐years) were observed among RASi users. In the comparative analysis of clinical outcomes, patients who maintained RASi therapy after initiating potassium binders showed a lower cumulative incidence of all‐cause hospitalization, all‐cause mortality and 3P‐MACE over the 3‐year follow‐up compared with those who discontinued or down‐titrated RASi therapy (Fig. S3). These differences remained consistent for hospitalization and all‐cause mortality after propensity score weighting (Fig. 2a, Fig. S4; HR: 0.77 [95% CI 0.72–0.83] and HR: 0.77 [95% CI 0.68–0.87], respectively), with no evidence of residual covariate imbalance (Fig. S5). In contrast, weighted cumulative incidence curves for 3P‐MACE largely overlapped, indicating no substantial differences between groups (Fig. 2a).

Results from sensitivity analyses using multiple imputation for missing baseline eGFR and potassium were consistent with the main findings (inpatient hospitalization HR: 0.77 [95% CI: 072–0.83]; 3P‐MACE HR: 0.85 [0.72–1.00]; all‐cause mortality HR: 0.77 [0.68–0.87]; Fig. S6). Similarly, estimates remained robust when redefining changes in RASi treatment status at 4 months rather than 6 months (Fig. S7).

In the comparison between first‐ and second‐generation potassium binders, no clear differences in outcome incidence were observed (Fig. 2b). In a subgroup analysis restricted to patients who maintained RASi therapy, second‐generation binder users showed numerically lower event rates than those receiving first‐generation binders, but the overlapping confidence intervals indicate no evidence of a difference (Fig. 2c).

In a supplementary analysis among patients who either discontinued or down‐titrated RASi, the pattern was reversed. Here, numerically higher event rates were observed in the second‐generation group, although these differences also lacked statistical significance (Fig. S8). Corresponding analyses of clinical outcomes were conducted for MRA treatment, but no evidence of differences were observed across the comparison groups (Figs. S9 and S10).

In supplementary analyses of hospitalization and mortality patterns, CKD Stage 5 was the most frequently recorded primary diagnosis at the time of inpatient admission, accounting for 12.1% of hospitalizations among patients with baseline RASi and/or MRA therapy, followed by HF (6.9%) (Table S1). Among recorded causes of death, HF was the most common (8.9%), followed by IHD (7.2%). Notably, coronavirus disease 2019 (COVID‐19) was the third most frequent cause of death, accounting for 7.0% of observed mortality cases (Table S2).

## Discussion

In this retrospective cohort study with patients having ND‐CKD, HF or both receiving RASi or MRA at the time of potassium binder episode, those treated with second‐generation potassium binders were more likely to maintain RASi or MRA therapy, compared with those treated with first‐generation potassium binders. Furthermore, patients who maintained RASi therapy following a potassium binder treatment episode had lower observed rates of all‐cause mortality and hospitalization than those who down‐titrated or discontinued. No differences in 3P‐MACE were observed between patient groups.

Hyperkalaemia is a major reason for underuse of guideline‐directed RAASi treatment in patients with CKD or HF. We found that patients with ND‐CKD, HF or both treated with second‐generation potassium binders (patiromer or sodium zirconium cyclosilicate) were more likely to maintain their RAASi treatment than those treated with first‐generation potassium binder SPS. Our results are in alignment with a previous study, where higher RAASi treatment maintenance was observed among those with hyperkalaemia and treated with a second‐generation potassium binder (patiromer) compared with those treated with first‐generation potassium binder (SPS) [24]. However, the current study is the first to compare RAASi treatment maintenance between the first‐generation potassium binder (SPS) and both second‐generation potassium binders (patiromer and sodium zirconium cyclosilicate) in patients with ND‐CKD, HF or both in Sweden.

Differences in patient characteristics were observed between those treated with first‐ versus second‐generation binders. Notably, those receiving the latter were younger, had a larger proportion of HF or HF and CKD, but a smaller proportion of CKD at baseline. A higher proportion of patients treated with second‐generation potassium binders remained on potassium binder therapy at 6 months post‐initiation compared with those receiving first‐generation agents. These results are in agreement with previous studies, including one utilizing the same database [16, 25]. However, it is important to note that the current study population only includes a specific subgroup of patients with prevalent ND‐CKD, HF or both.

Patients who maintained RASi therapy after treatment with potassium binders experienced lower rates of hospitalization and mortality during follow‐up. These associations persisted after propensity score weighting, though residual confounding may remain given the observational design and limited availability of lab‐based disease severity markers. In sensitivity analyses that additionally included baseline eGFR and potassium (with multiple imputation for missing values), results were consistent with the primary model, supporting the robustness of the observed associations. This aligns with previous research in those with CKD or HF, showing that the risk of adverse cardiorenal outcomes is increased if RAASi treatment is discontinued or down‐titrated after hyperkalaemia [11, 26, 27, 28]. Although earlier studies have reported that the continuation of MRA treatment is also associated with improved clinical outcomes [29, 30], we did not observe a difference in clinical outcomes based on MRA maintenance. Trevisan et al. observed differences in all‐cause mortality and MACE based on MRA continuation in patients with HF and hyperkalaemia [29]. However, despite their analysis, including more patients than our study, the reported absolute risks for adverse outcomes had overlapping confidence intervals similar to our results. This may suggest that our findings reflect limited statistical power. In contrast to lower rates of mortality and hospitalizations, we did not observe the reduced incidence of 3P‐MACE in those who maintained RASi therapy. This indicates that the observed lower rates of hospitalisations and deaths were mainly due to differences in outcomes not captured in the definition of the composite outcome 3P‐MACE.

Among patients that maintained RASi therapy, those treated with second‐generation potassium binders tended to have lower incidence of adverse clinical outcomes than those treated with first‐generation potassium binders. Unexpectedly, among patients who discontinued or down‐titrated RASi therapy, those receiving second‐generation potassium binders exhibited numerically higher adverse clinical outcomes compared with the first‐generation group. Although this is most likely due to the limited number of patients and events within this subgroup, leading to insufficient statistical power, residual confounding cannot be excluded. In addition, patients in this group may have had a more complex clinical profiles not fully captured by the measured covariates, despite weighting on propensity score.

The current study includes patients with a recorded potassium binder treatment episode between 2018 and 2022. This time frame was chosen because, although the first‐generation potassium binder SPS has been used in Sweden for several decades, second‐generation binders only became available in 2018. We found that a minority of the potassium binder treatment episodes in our study involved a second‐generation binder. This aligns with previous results and may reflect prescription inertia as well as the substantially lower cost of first‐generation potassium binders. Given that second‐generation potassium binders were associated with higher observed RAASi therapy persistence, which in turn was associated with improved clinical outcomes, these findings raise the question whether second generation binders should be used when managing hyperkalemia for patients with ND‐CKD and/or HF receiving RAASi treatment. However, it is important to note that we did not find clear differences in clinical outcomes between binder generations. Further studies with larger datasets are needed to better investigate associations between the choice of potassium binder generation, RAASi treatment decisions and clinical outcomes in this patient population.

The period analysed in the current study overlapped substantially with the COVID‐19 pandemic, during which the safety of using RAASi was questioned [31]. Although no evidence exists of RAASi impacting clinical outcomes of COVID‐19, this uncertainty may have influenced clinical decision‐making and RAASi prescription patterns [31, 32, 33]. However, assuming the COVID‐19 pandemic did not affect the choice of potassium binder generation, it is unlikely to explain differences in RAASi therapy maintenance observed between first‐ and second‐generation potassium binders. In addition, COVID‐19 was the third leading cause of death after HF and chronic IHD, in agreement with previous studies showing that both CKD and HF are risk factor for severe COVID‐19 [34, 35].

The strengths of this study include the use of a comprehensive electronic health data with virtually complete coverage of data from a well‐defined European healthcare setting with universal access to healthcare. In addition, this study includes an analysis of real‐world data to provide guidance for the management of hyperkalemia and use of potassium binders in patients with ND‐CKD and/or HF who are on RASi or MRA treatment. The results of the current study may be generalizable to other populations with universally accessible healthcare, as well as similar healthcare structures, patient baseline characteristics and prescribing patterns.

Despite the major strengths of this study, there are limitations to acknowledge. First, our study dataset also had a low number of treatment episodes with second‐generation potassium binders as well as MRA, which may have been insufficient to detect meaningful differences in some of the outcomes. In addition, the estimation of RAASi treatment changes relied on drug dispensation data from the Swedish Prescribed Drug Register, capturing medication supply rather than actual consumption. As a result, the misclassification of exposure may have occurred due to patient non‐adherence or temporary dose adjustments made verbally by the prescribing physician. To mitigate this, methodological adjustments were carried out to account for residual supply and imperfect adherence. Although the misclassification of RAASi exposure may still have occurred, the difference is likely non‐differential between potassium binder generations.

Furthermore, to evaluate RAASi persistence, we restricted the analysis to episodes with a medical supply of RAASi at index. Although this approach ensures that all treatment changes are measured from a common point of exposure, it may introduce selection toward patients with higher adherence or more active clinical management at baseline. This may represent a study somewhat healthier population or more treatment‐engaged subgroup. In addition, due to the requirement of at least 180 days of follow‐up, our findings may preferentially include healthier patients. Although covariate balancing was carried out when comparing clinical outcomes, there may still be possible residual confounding from unmeasured clinical or socioeconomic factors.

In conclusion, this real‐world, observational study found that, compared with the first‐generation potassium binder SPS, use of second‐generation potassium binders was associated with improved continuation of RASi and MRA therapy in patients with ND‐CKD, HF or both. In addition, the maintenance of RASi therapy was associated with lower observed rates of hospitalization and all‐cause mortality. These findings underscore the potential of potassium binders to optimize RAASi use in patients with ND‐CKD and/or HF.

## Author contributions

All authors fulfil the ICMJE requirements for authorship. **Hans Furuland**: Conceptualization (equal); investigation (equal); methodology (supporting); writing—original draft (equal); writing—review and editing (equal). **Anders Olof Larsson**: Conceptualization (equal); investigation (equal); methodology (supporting); writing—original draft (equal); writing—review and editing (equal). **Milica Uhde**: Conceptualization (equal); funding acquisition (lead); investigation (equal); methodology (supporting); writing—original draft (equal); writing—review and editing (equal). **Kristel Parv**: Conceptualization (equal); investigation (equal); visualization (supporting); writing—original draft (lead); writing—review and editing (equal). **Matilda Almstedt**: Conceptualization (equal); investigation (equal); project administration (lead); writing—original draft (equal); writing—review and editing (equal). **Thomas Cars**: Conceptualization (equal); data curation and formal analysis (lead); investigation (equal); methodology (lead); project administration (supporting); resources (lead); software (lead); validation (lead); visualization (lead); writing—original draft (equal); writing—review and editing (equal). **Maria K. Svensson**: Conceptualization (equal); investigation (equal); methodology (supporting); resources (lead); supervision (lead); writing—original draft (equal); writing—review and editing (equal).

## Conflict of interest statement

**Hans Furuland**: consultancy fees from AstraZeneca, GSK, Vifor Pharma Nordiska AB, Bayer, BMS Sanofi and Amgen. **Anders Olof Larsson**: no conflicts of interest to declare. **Milica Uhde**: employee at Vifor Pharma Nordiska AB with RSU. **Matilda Almstedt and Thomas Cars**: employees and shareholders at Sence Research AB, which is a company in biostatistics and epidemiology that received payment from Vifor Pharma Nordiska AB, Sweden for project management, data management and statistical analysis during the conduct of the study. **Kristel Parv**: employee at Sence Research AB. **Maria K Svensson**: consultancy fees from Sang Biotech AB; received payment for presentations/educational events/lectures/presentations from Amgen, AstraZeneca, Boehringer Ingelheim, Vifor Pharma Nordiska AB, GSK and Novo Nordisk.

## Funding information

This study was funded by Vifor Pharma Nordiska AB.

## Supporting information




**Figure S1**: Derivation of the study cohort.
**Figure S2**: Proportion of patients remaining on RASi and MRA therapy before and after initiation of potassium binder treatment.
**Figure S3**: Unweighted cumulative incidence of clinical outcomes stratified by (a) change in RASi treatment, (b) potassium binder generation, and (c) combined categories of RASi treatment change and potassium binder generation.
**Figure S4**: Propensity score diagnostics.
**Figure S5**: (a) Covariate balance before and after weighting among patients on baseline RASi or MRA, comparing those who maintained treatment versus those who down‐titrated or discontinued. (b) Covariate balance before and after weighting by potassium binder generation (first vs. second) among patients on baseline RASi or MRA. (c) Covariate balance before and after weighting comparing patients on first‐generation potassium binders with maintained RASi or MRA to those on second‐generation binders with maintained RASi or MRA.
**Figure S6**: (a) Propensity score diagnostics and (b) covariate balance before and after weighting for the sensitivity analysis including baseline eGFR and serum potassium in the propensity score mode.
**Figure S7**: Weighted cumulative incidence of all‐cause mortality stratified by change in RASi treatment (sensitivity analysis with a four‐month classification window for RASi therapy estimation).
**Figure S8**: Weighted cumulative incidence of clinical outcomes among those who down‐titrated/discontinued RASi/MRA stratified by strata of potassium binder generation.
**Figure S9**: Weighted cumulative incidence of clinical outcomes stratified by (a) change in MRA treatment, (b) potassium binder generation, and (c) combined categories of MRA treatment change and potassium binder generation.
**Figure S10**: Unweighted cumulative incidence of clinical outcomes stratified by (a) change in MRA treatment, (b) potassium binder generation, and (c) combined categories of MRA treatment change and potassium binder generation.
**Table S1**: Ten most common primary causes of inpatient hospitalization during follow‐up among patients with baseline RASi and/or MRA therapy.
**Table S2**: Ten most common primary causes of death among patients with baseline RASi and/or MRA therapy.

## Data Availability

The data that support the findings of this study are available upon reasonable request to MKS.

## References

[1] Massicotte‐Azarniouch D , Canney M , Sood MM , Hundemer GL . Managing hyperkalemia in the modern era: a case‐based approach. Kidney Int Rep. 2023;8:1290–300. 37441466 10.1016/j.ekir.2023.04.016 PMC10334407

[2] Chang AR , Sang Y , Leddy J , Yahya, T , Kirchner, HL , Inker, LA , et al. Antihypertensive medications and the prevalence of hyperkalemia in a large health system. Hypertens Dallas Tex 1979. 2016;67: 1181–8. 10.1161/HYPERTENSIONAHA.116.07363 PMC4865437 27067721

[3] Nilsson E , Gasparini A , Ärnlöv J , Xu H , Henriksson KM , Coresh J , et al., Incidence and determinants of hyperkalemia and hypokalemia in a large healthcare system. Int J Cardiol. 2017;245:277–84. 28735756 10.1016/j.ijcard.2017.07.035

[4] Murphy D , Banerjee D . Hyperkalaemia in heart failure: consequences for outcome and sequencing of therapy. Curr Heart Fail Rep. 2022;19:191–9. 35704263 10.1007/s11897-022-00552-3 PMC9329160

[5] Stevens PE , Ahmed SB , Carrero JJ , Foster B , Francis A , Hall RK , et al., KDIGO 2024 clinical practice guideline for the evaluation and management of chronic kidney disease. Kidney Int. 2024;105:S117–314. 38490803 10.1016/j.kint.2023.10.018

[6] McDonagh TA , Metra M , Adamo M , Gardner RS , Baumbach A , Böhm M , et al., 2021 ESC guidelines for the diagnosis and treatment of acute and chronic heart failure. Eur Heart J. 2021;42:3599–726. 34922348 10.1093/eurheartj/ehab853

[7] Palmer BF , Carrero JJ , Clegg DJ , Colbert GB , Emmett M , Fishbane S , et al., Clinical management of hyperkalemia. Mayo Clin Proc. 2021;96:744–62. 33160639 10.1016/j.mayocp.2020.06.014

[8] Weinstein J , Girard LP , Lepage S , McKelvie RS , Tennankore K . Prevention and management of hyperkalemia in patients treated with renin‐angiotensin‐aldosterone system inhibitors. CMAJ Can Med Assoc J J Assoc Medicale Can. 2021;193:E1836–41. 10.1503/cmaj.210831 PMC8648362 34872955

[9] Agarwal R , Rossignol P , Romero A , Garza D , Mayo MR , Warren S , et al., Patiromer versus placebo to enable spironolactone use in patients with resistant hypertension and chronic kidney disease (AMBER): a phase 2, randomised, double‐blind, placebo‐controlled trial. The Lancet. 2019;394:1540–50. 10.1016/S0140-6736(19)32135-X 31533906

[10] Hattori K , Sakaguchi Y , Oka T , Asahina Y , Kawaoka T , Doi Y , et al., Estimated effect of restarting renin‐angiotensin system inhibitors after discontinuation on kidney outcomes and mortality. J Am Soc Nephrol. 2024;35:1391–401. 38889205 10.1681/ASN.0000000000000425 PMC11452132

[11] Kanda E , Rastogi A , Murohara T , Lesén E , Agiro A , Arnold M , et al., Clinical impact of suboptimal RAASi therapy following an episode of hyperkalemia. BMC Nephrol. 2023;24:18. 36658531 10.1186/s12882-022-03054-5 PMC9854063

[12] Sterns RH , Grieff M , Bernstein PL . Treatment of hyperkalemia: something old, something new. Kidney Int. 2016;89:546–54. 26880451 10.1016/j.kint.2015.11.018

[13] Butler J , Anker SD , Lund LH , Coats AJS , Filippatos G , Siddiqi TJ , et al., Patiromer for the management of hyperkalemia in heart failure with reduced ejection fraction: the DIAMOND trial. Eur Heart J. 2022;43:4362–73. 35900838 10.1093/eurheartj/ehac401 PMC9622299

[14] Pitt B , Anker SD , Bushinsky DA , Kitzman DW , Zannad F , Huang I‐Z , Evaluation of the efficacy and safety of RLY5016, a polymeric potassium binder, in a double‐blind, placebo‐controlled study in patients with chronic heart failure (the PEARL‐HF) trial. Eur Heart J. 2011;32:820–8. 21208974 10.1093/eurheartj/ehq502 PMC3069389

[15] Spinowitz BS , Fishbane S , Pergola PE , Roger SD , Lerma EV , Butler J , et al., Sodium zirconium cyclosilicate among individuals with hyperkalemia: a 12‐month phase 3 study. Clin J Am Soc Nephrol CJASN. 2019;14:798–809. 31110051 10.2215/CJN.12651018 PMC6556727

[16] Furuland H , Larsson AO , Bjellerup P , Uhde M , Cars T , Almstedt M , et al., Potassium binders in clinical practice: understanding potassium binder use in contemporary Swedish healthcare‐the DEMONSTRATE database. BMC Nephrol. 2025;26:213. 40295946 10.1186/s12882-025-04146-8 PMC12036272

[17] Ludvigsson JF , Bergman D , Lundgren CI , Sundquist K , Geijerstam JLA , Glenngård AH , et al., The healthcare system in Sweden. Eur J Epidemiol. 2025;40:563–579. 40383868 10.1007/s10654-025-01226-9 PMC12170770

[18] Wettermark B , Hammar N , MichaelFored C , Leimanis A , Otterblad Olausson P , Bergman U , et al., The new swedish prescribed drug register–opportunities for pharmacoepidemiological research and experience from the first six months. Pharmacoepidemiol Drug Saf. 2007;16:726–35. 16897791 10.1002/pds.1294

[19] Brooke HL , Talbäck M , Hörnblad J , Johansson LA , Ludvigsson JF , Druid H , et al., The Swedish cause of death register. Eur J Epidemiol. 2017;32:765–73. 28983736 10.1007/s10654-017-0316-1 PMC5662659

[20] World Health Organization . International statistical classification of diseases and related health problems, 10th revision. WHO; 2019.

[21] WHO Collaborating Centre for Drug Statistics Methodology . ATC classification index with DDDs. WHOCC; 2020.

[22] Lee BK , Lessler J , Stuart EA . Weight trimming and propensity score weighting. PLoS ONE. 2011;6:e18174. 21483818 10.1371/journal.pone.0018174 PMC3069059

[23] Austin PC . An introduction to propensity score methods for reducing the effects of confounding in observational studies. Multivar Behav Res. 2011;46:399–424. 10.1080/00273171.2011.568786 PMC3144483 21818162

[24] Desai NR , Rowan CG , Alvarez PJ , Fogli J , Toto RD . Hyperkalemia treatment modalities: a descriptive observational study focused on medication and healthcare resource utilization. PLoS ONE. 2020;15:e0226844. 31910208 10.1371/journal.pone.0226844 PMC6946143

[25] Gonzalez‐Ortiz A , Clase CM , Bosi A , Fu EL , Pérez‐Guillé BE , Faucon A‐L , et al., Evaluation of the introduction of novel potassium binders in routine care; the Stockholm CREAtinine measurements (SCREAM) project. J Nephrol. 2024;37:961–72. 38236474 10.1007/s40620-023-01860-0 PMC11239771

[26] Leon SJ , Whitlock R , Rigatto C , Komenda P , Bohm C , Sucha E , et al., Hyperkalemia‐related discontinuation of renin‐angiotensin‐aldosterone system inhibitors and clinical outcomes in CKD: a population‐based cohort study. Am J Kidney Dis. 2022;80:164–173.e1. 35085685 10.1053/j.ajkd.2022.01.002

[27] Xu Y , Fu EL , Trevisan M , Jernberg T , Sjölander A , Clase CM , et al., Stopping renin‐angiotensin system inhibitors after hyperkalemia and risk of adverse outcomes. Am Heart J. 2022;243:177–86. 34610282 10.1016/j.ahj.2021.09.014

[28] Svensson MK , Fischereder M , Kalra PR , Sánchez Lázaro IJé , Lesén E , et al., Estimated number needed to treat to avoid a first hospitalization by maintaining instead of reducing renin‐angiotensin‐aldosterone system inhibitor (RAASi) Therapy after hyperkalemia. Kidney360. 2024;5:1813–23. 39167454 10.34067/KID.0000000000000561 PMC11687985

[29] Trevisan M , Fu EL , Xu Y , Savarese G , Dekker FW , Lund LH , et al., Stopping mineralocorticoid receptor antagonists after hyperkalaemia: Trial emulation in data from routine care. Eur J Heart Fail. 2021;23:1698–707. 34196082 10.1002/ejhf.2287

[30] Rossignol P , Lainscak M , Crespo‐Leiro MG , Laroche Cé , Piepoli MF , Filippatos G , et al., Unravelling the interplay between hyperkalaemia, renin–angiotensin–aldosterone inhibitor use and clinical outcomes. Data from 9222 chronic heart failure patients of the ESC‐HFA‐EORP heart failure long‐term registry. Eur J Heart Fail. 2020;22:1378–89. 32243669 10.1002/ejhf.1793

[31] Loader J , Lampa E , Gustafsson S , Cars T , Sundström J . Renin‐angiotensin aldosterone system inhibitors in primary prevention and COVID‐19. J Am Heart Assoc. 2021;10:e021154. 34320843 10.1161/JAHA.120.021154 PMC8475700

[32] Gnanenthiran SR , Borghi C , Burger D , Caramelli B , Charchar F , Chirinos JA , et al., Renin‐angiotensin system inhibitors in patients with COVID‐19: a meta‐analysis of randomized controlled trials led by the international society of hypertension. J Am Heart Assoc. 2022;11:e026143. 36000426 10.1161/JAHA.122.026143 PMC9496439

[33] Guscoth L , Hodgson S . Analysis of the trend in community prescribing of RAAS inhibitors during the COVID‐19 pandemic. Br J Cardiol. 2021;28:44. 35747072 10.5837/bjc.2021.044 PMC9063701

[34] Artborg A , Caldinelli A , Wijkström J , Nowak A , Fored M , Stendahl M , et al., Risk factors for COVID‐19 hospitalization and mortality in patients with chronic kidney disease: a nationwide cohort study. Clin Kidney J. 2024;17:sfad283. 38186903 10.1093/ckj/sfad283 PMC10768790

[35] Ritsinger V , Bodegård J , Kristofi R , Thuresson M , Nathanson D , Nyström T , et al., History of heart failure and chronic kidney disease and risk of all‐cause death after COVID‐19 during the first three waves of the pandemic in comparison with influenza outbreaks in Sweden: a registry‐based, retrospective, case‐control study. BMJ Open. 2023;13:e069037. 10.1136/bmjopen-2022-069037 PMC10151240 37117003

