# Supplementary material for: Potassium binders and continuation of renin–angiotensin system inhibitors/mineralocorticoid receptor antagonist in chronic kidney disease and heart failure (the DEMONSTRATE database)
Source: J Intern Med. 2026 Mar 26;300(2):179–92. doi: 10.1111/joim.70087 (PMC13327437; doi:10.1111/joim.70087)
Supplement: Supplementary file 1 — Figure S1: Derivation of the study cohort. Figure S2: Proportion of patients remaining on RASi and MRA therapy before and after initiation of potassium binder treatment. Figure S3: Unweighted cumulative incidence of clinical outcomes stratified by (a) change in RASi treatment, (b) potassium binder generation, and (c) combined categories of RASi treatment change and potassium binder generation. Figure S4: Propensity score diagnostics. Figure S5: (a) Covariate balance before and after weighting among patients on baseline RASi or MRA, comparing those who maintained treatment versus those who down‐titrated or discontinued. (b) Covariate balance before and after weighting by potassium binder generation (first vs. second) among patients on baseline RASi or MRA. (c) Covariate balance before and after weighting comparing patients on first‐generation potassium binders with maintained RASi or MRA to those on second‐generation binders with maintained RASi or MRA. Figure S6: (a) Propensity score diagnostics and (b) covariate balance before and after weighting for the sensitivity analysis including baseline eGFR and serum potassium in the propensity score mode. Figure S7: Weighted cumulative incidence of all‐cause mortality stratified by change in RASi treatment (sensitivity analysis with a four‐month classification window for RASi therapy estimation). Figure S8: Weighted cumulative incidence of clinical outcomes among those who down‐titrated/discontinued RASi/MRA stratified by strata of potassium binder generation. Figure S9: Weighted cumulative incidence of clinical outcomes stratified by (a) change in MRA treatment, (b) potassium binder generation, and (c) combined categories of MRA treatment change and potassium binder generation. Figure S10: Unweighted cumulative incidence of clinical outcomes stratified by (a) change in MRA treatment, (b) potassium binder generation, and (c) combined categories of MRA treatment change and potassium binder generation. Table S1: Ten most com [file JOIM-300-179-s001.docx]

Supplementary Methods

Definition of potassium binder episode

For any given patient, the first dispensation of a potassium binder during the study period marked the start of their first potassium binder treatment episode. If a subsequent dispensation occurred within ≤180 days of the previous one, the episode was extended by an additional 180 days. Thus, a single treatment episode could encompass multiple dispensations. If no further dispensation occurred within 180 days of the previous one, the treatment episode was considered ended. A new potassium binder treatment episode was initiated if any subsequent dispensations occurred thereafter.


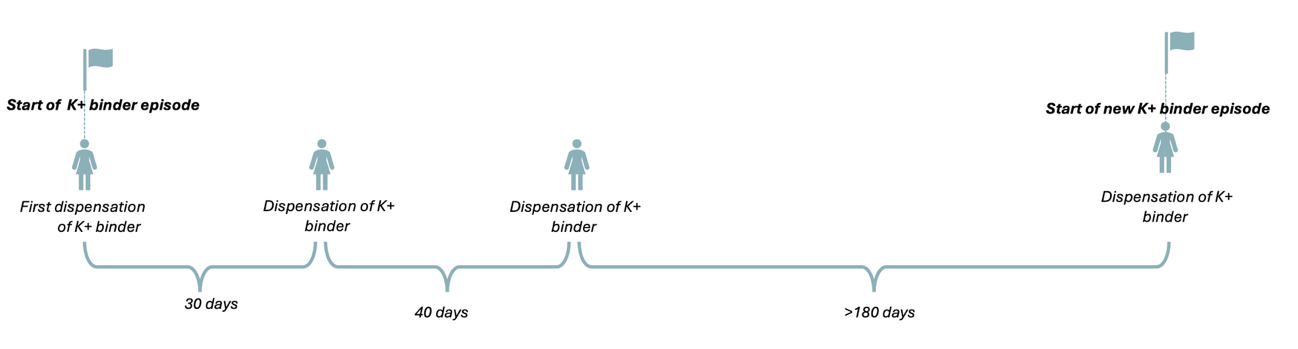


Definition of prevalent comorbidities

Listed below are the ICD-10 codes and clinical procedure codes used to identify prevalent comorbidities of interest in each patient. The codes listed below were used to identify prevalent conditions for each patient. Patients were classified as having a condition if a corresponding record was found at any time prior to or on the index date.

| Diagnosis or procedure | ICD-10 codes | Procedure codes |
| --- | --- | --- |
| Heart failure | I50, I11.0, I13.0, I13.2, I25.5, I42.0, I42.6, I42.7, I42.9, I43.1 |  |
| Chronic kidney disease | E10.2, E11.2, E12.2, E13.2, E14.2, N08.3, N18.X, I12.0, I12.9, I13.1, I13.2, I13.9, Z49.1, Z49.2, Z99.2 | JAK10, TJA33, TJA35, DJ008, DR013, DR014, DR015, DR016, DR017, DR023, DR024, DR055, DR056, DR060, DR061, KAS10, KAS20, QF006 |
| Hypertension | I10, I11, I12, I13, I15 |  |
| Dialysis | Z49.1, Z49.2, Z99.2 | DR013, DR014, DR015, DR016, DR017, DR023, DR024, DR055, DR056, DR060, DR061 |

Diabetes Mellitus

Patients with diabetes mellitus often have records of both type I and type II diabetes during their disease history. To enable a valid and mutually exclusive categorization, patients were classified as having type I or type II diabetes based on the following procedure:


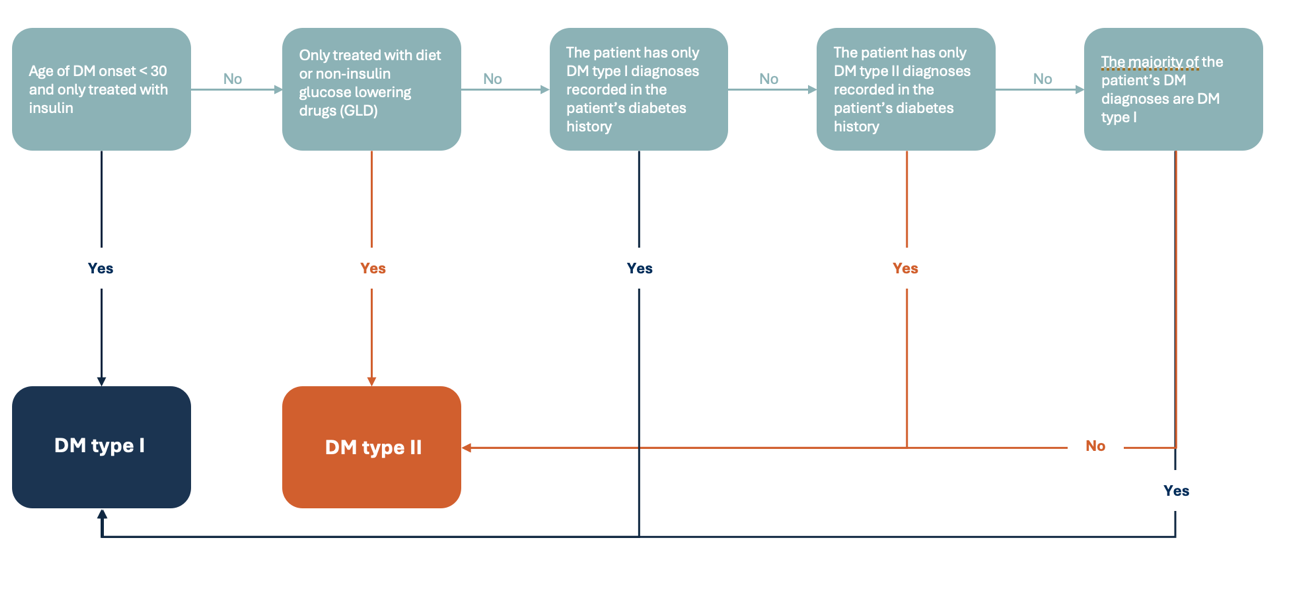


Definition of drug utilisation

Listed below are the ATC codes used to identify medication use of interest by each patient during the 180 days prior to and on the index date.

| Medication | ATC codes |
| --- | --- |
| RASi (ACEi/ARB/ARNi) | C09 |
| RAASi (ACEi, ARB, ARNi, MRA) | C09, C03DA |
| MRA | C03DA |
| Antihypertensives | C02, C09, C03A, C07, C08C |
| Diuretics | C03A, C03B, C03C, C03E |
| Beta blockers | C07 |
| Calcium channel blockers | C08C |
| SGLT2i | A10BK, A10DB15, A10DB16, A10DB19, A10DB20, A10DB21, A10DB23, A10DB24 |

Abbreviations: ACEi, angiotensin-converting enzyme inhibitors; ARB, angiotensin receptor blockers; ARNi, angiotensin receptor-neprilysin inhibitors; MRA, mineralocorticoid receptor antagonists; RAASi, renin-angiotensin aldosterone system inhibitors; RASi, renin-angiotensin system inhibitors; SGLT2i, sodium-glucose cotransporter 2 inhibitors.

Algorithm to define RAASi changes

Changes in renin-angiotensin-aldosterone system (RAAS) inhibitor use were analysed separately for angiotensin-converting enzyme inhibitors (ACEi)/angiotensin receptor blockers (ARB) and mineralocorticoid receptor antagonists (MRA). These analyses were restricted to patients with at least 180 days of follow-up and to potassium binder episodes where ACEi/ARB or MRA were used at the time of potassium binder initiation (index date). Treatment status at the index date was determined based on the most recent pharmacy dispensation of ACEi/ARB and MRA prior to the index date.

For each potassium binder episode, changes in ACEi/ARB and MRA treatment were assessed at 180 post-index. If a pharmacy dispensation provided medication coverage up to day 180, the episode was classified as still being treated with ACEi/ARB or MRA. If coverage did not extend to day 180, the episode was categorized as discontinued. To evaluate changes in dose, all prescribed doses were converted to a proportion of the target dose for each substance. For example, a prescribed dose of 50 mg of Losartan was considered 33.3% of the target dose, as the target dose for Losartan is 150 mg.

The following changes were assessed:

- Maintained RASi (ACEi/ARB) or MRA: Defined as no observed change in the proportion of the target dose.
- Decreased RASi (ACEi/ARB) or MRA: Defined as a reduction in the proportion of the target dose.
- Increased RASi (ACEi/ARB) or MRA: Defined as an increase in the proportion of the target dose.
- Discontinued RASi (ACEi/ARB) or MRA: Defined as no medical supply observed at day 180 post-index.

The following target doses have been used:

| ATC code | Substance name | Target dose |
| --- | --- | --- |
| C09AA02 | Enalapril | 20 mg |
| C09CA01 | Losartan | 150 mg |
| C09CA06 | Candesartan | 32 mg |
| C03DA01 | Spironolactone | 50 mg |
| C09AA04 | Perindopril | 10 mg |
| C09AA05 | Ramipril | 10 mg |
| C09CA04 | Irbesartan | 300 mg |
| C09DX04 | Valsartan and Sacubitril | 320 mg |
| C03DA04 | Eplerenone | 50 mg |
| C09DA06 | Candesartan and diuretics | 32 mg |
| C09BA02 | Enalapril and diuretics | 20 mg |
| C09DA01 | Losartan and diuretics | 150 mg |
| C09CA03 | Valsartan | 320 mg |
| C09AA03 | Lisinopril | 20 mg |
| C09CA07 | Telmisartan | 80 mg |
| C09DA04 | Irbesartan and diuretics | 300 mg |
| C09DA03 | Valsartan and diuretics | 320 mg |
| C09AA01 | Captopril | 150 mg |
| C09BA05 | Ramipril and diuretics | 10 mg |
| C09DA07 | Telmisartan and diuretics | 80 mg |
| C09DB01 | Valsartan and amlodipine | 320 mg |
| C09BA03 | Lisinopril and diuretics | 20 mg |
| C09CA02 | Eprosartan | 600 mg |
| C09BA08 | Cilazapril and diuretics | 5 mg |
| C09AA09 | Fosinopril | 20 mg |
| C09BA06 | Quinapril and diuretics | 20 mg |
| C09AA08 | Cilazapril | 2.5 mg |
| C09AA06 | Quinapril | 20 mg |
| C09AA10 | Trandolapril | 2 mg |
| C09DA02 | Eprosartan and diuretics | 600 mg |
| C03DA05 | Finerenone | 20 mg |

Supplementary Figures

Supplementary figure 1. Derivation of the study cohort


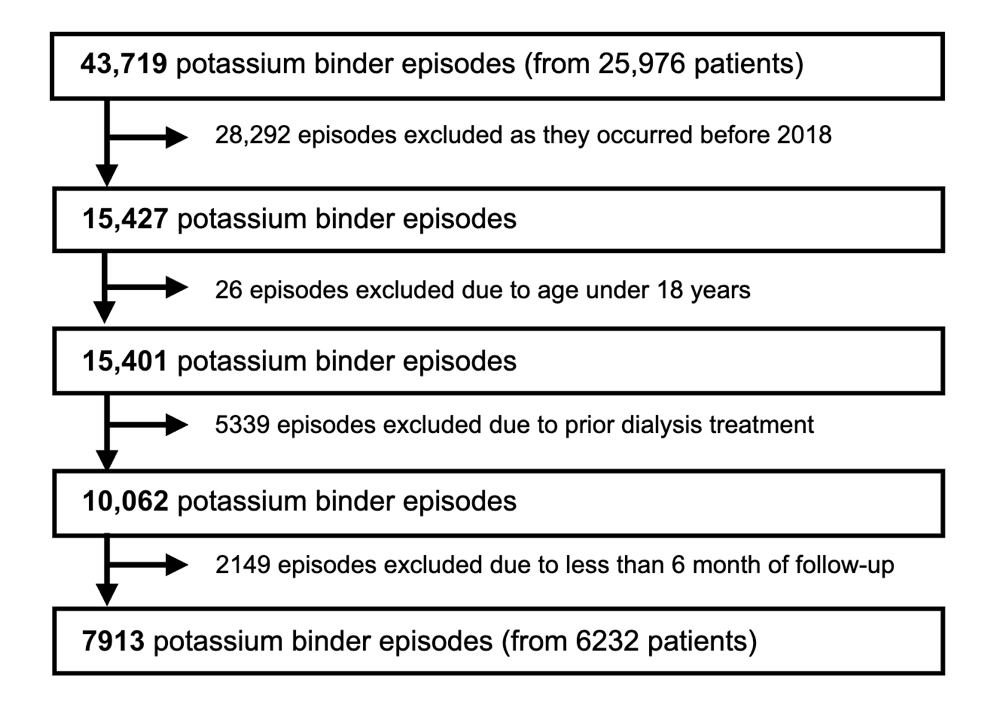


Supplementary figure 2. Proportion of patients remaining on RASi and MRA therapy before and after initiation of potassium binder treatment

Abbreviations: MRA, mineralocorticoid receptor antagonist; RASi, renin-angiotensin system inhibitor.

Supplementary Figure 3. Unweighted cumulative incidence of clinical outcomes stratified by (a) change in RASi treatment, (b) potassium binder generation, and (c) combined categories of RASi treatment change and potassium binder generation

Unweighted Kaplan-Meier estimates for clinical outcomes among episodes with baseline RASi use. Abbreviations: MACE, major adverse cardiovascular event; RASi, renin-angiotensin system inhibitor.

Supplementary Figure 4. Propensity score diagnostics.

Density plots showing the distribution of propensity scores stratified by a) treatment changes, b) potassium binder generation in those treated with either RASi or MRA at baseline, c) potassium binder generation among those that maintained RASi treatment, and d) potassium binder generation among those that maintained MRA

Supplementary Figure 5. Covariate balance

To evaluate covariate balance before and after weighting, standardized mean differences (SMDs) were calculated for all treatment comparisons. An SMD ± 0.1 SMD (indicated by vertical dashed lines) was interpreted as evidence of negligible imbalance. In the outcome analyses, three treatment comparisons were conducted for each of RASi and MRA, resulting in a total of six covariate balance assessments.

Supplementary Figure 5a. Covariate balance before and after weighting among patients on baseline RASi or MRA, comparing those who maintained treatment versus those who down-titrated or discontinued

Abbreviations: CCB, calcium channel blocker; CKD, chronic kidney disease; IHD, ischemic heart disease; MRA, mineralocorticoid receptor antagonist; RASi, renin-angiotensin system inhibitor; SGLT2i, sodium-glucose cotransporter-2 inhibitors.

Supplementary Figure 5b. Covariate balance before and after weighting by potassium binder generation (first vs. second) among patients on baseline RASi or MRA

Abbreviations: CCB, calcium channel blocker; CKD, chronic kidney disease; IHD, ischemic heart disease; MRA, mineralocorticoid receptor antagonist; RASi, renin-angiotensin system inhibitor; SGLT2i, sodium-glucose cotransporter-2 inhibitors.

Supplementary Figure 5c. Covariate balance before and after weighting comparing patients on first-generation potassium binders with maintained RASi or MRA to those on second-generation binders with maintained RASi or MRA

Abbreviations: CCB, calcium channel blocker; CKD, chronic kidney disease; HF, heart failure; IHD, ischemic heart disease; MRA, mineralocorticoid receptor antagonist; RASi, renin-angiotensin system inhibitor; SGLT2i, sodium-glucose cotransporter-2 inhibitors.

Supplementary figure 6. a) Propensity score diagnostics and b) covariate balance before and after weighting for the sensitivity analysis including baseline eGFR and serum potassium in the propensity score model

Covariate balance and propensity score distributions shown for the first imputed dataset (m = 1 of 10). Abbreviations: CCB, calcium channel blocker; CKD, chronic kidney disease; eGFR, estimated glomerular filtration rate; IHD, ischemic heart disease; MRA, mineralocorticoid receptor antagonist; SGLT2i, sodium-glucose cotransporter-2 inhibitors.

Supplementary figure 7. Weighted cumulative incidence of all-cause mortality stratified by change in RASi treatment (sensitivity analysis with a four-month classification window for RASi therapy estimation).

a) Propensity score diagnostics, b) covariate balance before and after weighting, and c) weighted cumulative incidence of all-cause mortality stratified by change in RASi treatment with a four-month classification window. Abbreviations: CCB, calcium channel blocker; CKD, chronic kidney disease; IHD, ischemic heart disease; MRA, mineralocorticoid receptor antagonist; RASi, renin-angiotensin system inhibitor; SGLT2i, sodium-glucose cotransporter-2 inhibitors.

Supplementary Figure 8. Weighted cumulative incidence of clinical outcomes among those who down-titrated/discontinued RASi/MRA stratified by strata of potassium binder generation.

a) Weighted cumulative incidence of clinical outcomes among those who down-titrated/discontinued RASi, with respective b) propensity score diagnostics and covariate balance before and after weighting. c) Weighted cumulative incidence of clinical outcomes among those who down-titrated/discontinued MRA, with respective d) propensity score diagnostics and covariate balance before and after weighting. Abbreviations: CCB, calcium channel blocker; CKD, chronic kidney disease; IHD, ischemic heart disease; MACE, major adverse cardiovascular event; MRA, mineralocorticoid receptor antagonist; RASi, renin-angiotensin system inhibitor; SGLT2i, sodium-glucose cotransporter-2 inhibitors.

Supplementary Figure 9. Weighted cumulative incidence of clinical outcomes stratified by (a) change in MRA treatment, (b) potassium binder generation, and (c) combined categories of MRA treatment change and potassium binder generation

Weighted Kaplan-Meier estimates for clinical outcomes among episodes with baseline MRA use. Abbreviations: MACE, major adverse cardiovascular event; MRA, mineralocorticoid receptor antagonist.

Supplementary Figure 10. Unweighted cumulative incidence of clinical outcomes stratified by (a) change in MRA treatment, (b) potassium binder generation, and (c) combined categories of MRA treatment change and potassium binder generation

Unweighted Kaplan-Meier estimates for clinical outcomes among episodes with baseline MRA use. Abbreviations: MACE, major adverse cardiovascular event; MRA, mineralocorticoid receptor antagonist.

Supplementary Tables

Supplementary Table 1. Ten most common primary causes of inpatient hospitalization during follow-up among patients with baseline RASi and/or MRA therapy

| ICD10 | Description | Proportion (%) |
| --- | --- | --- |
| N18.5 | Chronic kidney disease, stage 5 | 12.1% |
| I50.9 | Heart failure, unspecified | 6.9% |
| U07.1 | COVID-19, virus identified | 2.4% |
| Z49.0 | Preparatory care for dialysis | 2.4% |
| J18.9 | Pneumonia, unspecified | 1.8% |
| N17.9 | Acute renal failure, unspecified | 1.8% |
| N39.0 | Urinary tract infection, site not specified | 1.8% |
| N18.9 | Chronic kidney disease, unspecified | 1.6% |
| J15.9 | Bacterial pneumonia, unspecified | 1.5% |
| N10.9 | Acute tubulo-interstitial nephritis | 1.3% |

Supplementary Table 2. Ten most common primary causes of death among patients with baseline RASi and/or MRA therapy

| ICD10 | Description | Proportion (%) |
| --- | --- | --- |
| I50.9 | Heart failure, unspecified | 8.9% |
| I25.9 | Chronic ischaemic heart disease, unspecified | 7.2% |
| U07.1 | COVID-19, virus identified | 7.0% |
| N18.9 | Chronic kidney disease, unspecified | 5.8% |
| I21.9 | Acute myocardial infarction, unspecified | 4.3% |
| E11.2 | Diabetes Mellitus type II with renal complications | 3.3% |
| I48.2 | Chronic atrial fibrillation | 3.1% |
| E14.2 | Unspecified Diabetes Mellitus with renal complications | 3.1% |
| E14.7 | Unspecified Diabetes Mellitus with multiple complications | 2.9% |
| E11.7 | Diabetes Mellitus type II with multiple complications | 2.2% |
